# Texture-Differentiated Grain Growth in Silicon Steel: Experiments and Modeling

**DOI:** 10.3390/ma17123037

**Published:** 2024-06-20

**Authors:** Songtao Chang, Yuhui Sha, Gengsheng Cao, Fang Zhang, Liang Zuo

**Affiliations:** Key Laboratory for Anisotropy and Texture of Materials, Ministry of Education, Northeastern University, Shenyang 110819, China; cst_chang@163.com (S.C.); cgsformal94@163.com (G.C.); zhangf@smm.neu.edu.cn (F.Z.); lzuo@mail.neu.edu.cn (L.Z.)

**Keywords:** grain growth, texture, grain size distribution, modeling, silicon steel

## Abstract

Grain growth for various texture components in silicon steel was investigated via experiments and modeling. It was found that the clustered spatial arrangement of grains with specific orientations significantly altered the local environment for grain growth and consequently resulted in texture-differentiated grain size distribution (GSD) evolution. A novel local-field model was proposed to describe grain growth driven by continuous changing orientation and size distribution of adjacent grains. The modelling results show that the texture-differentiated grain growth in microstructure with grain clusters can produce a GSD with increased proportion in small-sized range and large-sized range by more than two-times, accompanied with an evident change in area fractions of various texture components. The effect of clustered spatial arrangement on grain growth can be precisely predicted, which is valuable to design and control the texture-differentiated GSD as well as the global GSD.

## 1. Introduction

Grain size has long been a subject of research due to its critical role on various properties of materials. The grain size is conventionally designated as the mean grain size irrespective of the dispersed nature of grain size population [1,2,3]. Actually, the properties of materials, such as yield strength [4], resistance to corrosion [5], and magnetic properties [6], are sensitive not only to the average grain size but also the grain size distribution (GSD). Moreover, recrystallization texture is an essential aspect of material characteristics responsible for property anisotropy. It is well-known that Goss ({110}<001>) and {100} are the ideal recrystallization texture in grain-oriented and non-oriented silicon steel, respectively [7,8]. Texture-differentiated GSD has been reported frequently in IF steels [9], low-carbon steel [10,11], and α-titanium [12]. The well-controlled GSD has become a determinant microstructure parameter in the production of high-performance metallic materials.

Recrystallization texture is developed by the competition among various components during nucleation and growth [13]. Grains with specific orientations with higher nucleation kinetics or growth rate will determine the texture formation by frequency and/or size advantages. Consequently, grain growth often exhibits a texture dependence at the completion of recrystallization [14,15] and during further grain growth [16,17]. While most studies have focused on texture content and average grain size [17,18,19,20,21], the GSD varying with texture components has rarely been characterized. Abbruzzese et al. [22] found in silicon steel that Goss grains obtain a significantly larger size and distribution width after grain growth with a similar initial GSD to matrix grains. Brandt et al. [23] also reported that γ (<111>//ND, normal direction) grains have a larger size and distribution width after grain growth despite an initial size disadvantage in an Fe-17%Cr alloy. The anisotropic grain growth may be attributed to the difference in grain boundary mobility.

Abbruzzese et al. [24,25] introduced the orientation-dependent grain boundary energy and mobility into Hillert’s mean-field model, and simulated different grain growths for various texture components. In the mean-field model, grains belonging to a specific component grow in a homogeneous equivalent medium (HEM) composed of all texture components. However, the grain spatial arrangement of any component is heterogeneous, which directly affects the growth environment and results in texture-differentiated GSD. Therefore, the spatial heterogeneity should be incorporated to describe the GSD evolution during grain growth in a textured microstructure.

In this paper, we proposed a novel local-field model for grain growth considering spatial heterogeneity, where each grain exchanges matter only with its adjacent grains. The model extends beyond the classic mean-field model by updating the adjacent grains based on the coordinates in space, so that heterogeneous texture-differentiated grain growth kinetics and GSD evolution can be predicted. This model was validated by grain growth measurement in silicon steel, and the grain growth involved with texture-related clusters was modeled and analyzed.

## 2. Materials and Methods

The studied materials were laboratory-made silicon steel sheets containing 0.003 wt.% C, 3.0 wt.% Si, 0.2 wt.%, Mn, 0.0007 wt.% S, 0.009 wt.% P and balance Fe. Primarily recrystallized sheets were annealed at 850 °C to investigate grain growth. Microstructure and texture of the primarily recrystallized and annealed samples were characterized by a Crossbeam-550 scanning electron microscope (Zeiss, Oberkochen, Germany) equipped with a Symmetry S3 electron backscatter diffraction (EBSD) system (Oxford Instruments, Oxford, Britain). The rolling direction–normal direction section was observed by EBSD with a step size of 1 μm. Some of the primary recrystallized samples characterized by EBSD were further annealed at 850 °C for 20 min in a vacuum tube furnace with vacuum < 1 × 10^−4^ Pa and analyzed by EBSD to track the in situ grain growth.

Grain boundaries with misorientation from 2° to 15° and more than 15° awee defined as low-angle boundaries and high-angle boundaries, respectively. Grains were identified based on the criterion of an average in-grain misorientation threshold less than 1°. A minimum of 9 pixels per grain was ensured to differentiate the smallest effective grain. The sectional grain size (hereafter referred to as grain size), represented by the equivalent area diameter, was calculated from the grain area measured by EBSD. Orientation distribution functions (ODFs) were calculated from EBSD data using a harmonic series expansion method with a Gaussian half-width of 5°. Texture components were defined within 15° tolerance of the exact orientation. The test area for the grain size distribution, average grain size, and ODFs was greater than 25 mm^2^ with more than 10,000 grains.

## 3. Experimental Results

### 3.1. Microstructure and Texture

**Figure 1:**
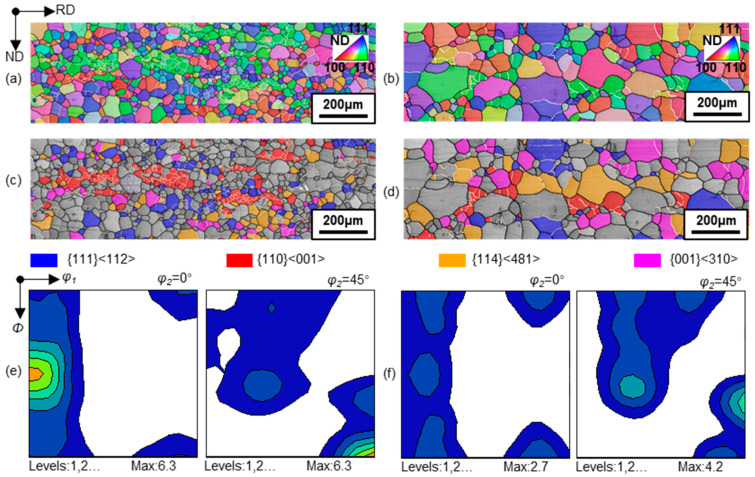
Orientation image maps and constant *φ*_2_ = 0° and 45° sections of ODFs in primarily recrystallized (**a**,**c**,**e**) and annealed (**b**,**d**,**f**) silicon steel sheets.

Figure 1 shows the microstructure of the selected representative regions and the ODFs. The primarily recrystallized sheet showed microstructural heterogeneity in the form of Goss grain clusters, and texture consisted of relatively strong η fiber with peak at Goss, γ fiber with peak at {111}<112> and weak λ (<001>//ND) fiber. After grain growth, the average grain size increased, while Goss grain clusters were consumed by adjacent grains. Goss texture decreased markedly, in contrast with the increase in {111}<112>, {114}<481> and {001}<310> texture components.

**Figure 2:**
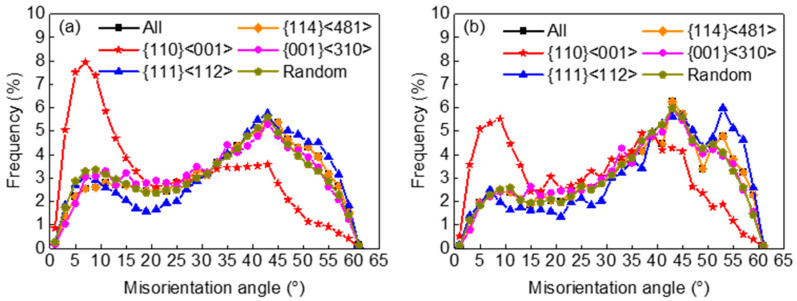
Grain boundary misorientation distributions for all grains and major texture components in primarily recrystallized (**a**) and annealed (**b**) silicon steel sheets.

The existence of a grain cluster significantly affected grain boundary misorientation distribution (GBMD). Figure 2 shows the GBMD of major texture components before and after grain growth. Goss grains possessed a higher proportion of low-angle grain boundaries, and the gap was somewhat reduced after grain growth.

**Figure 3:**
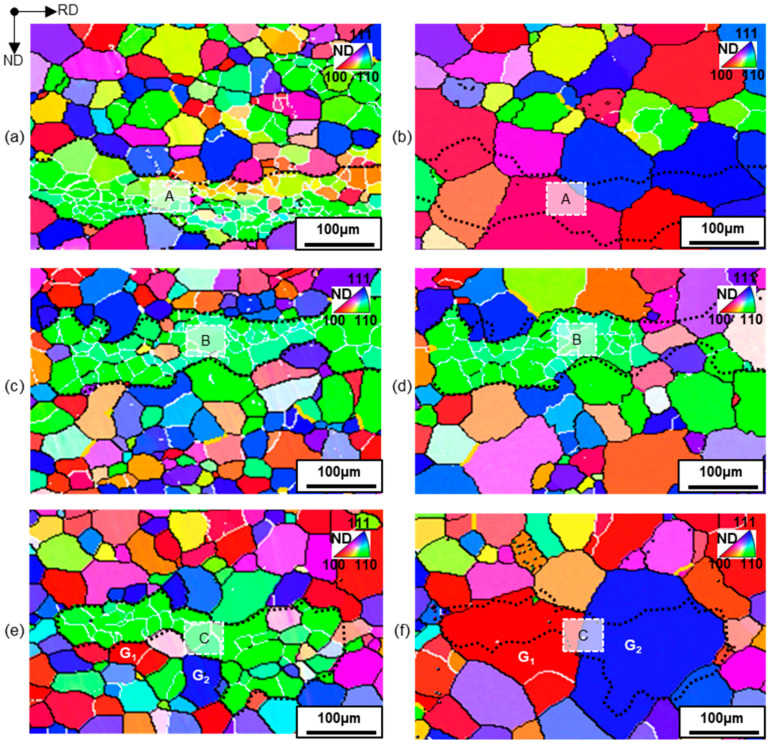
Orientation image maps of identical region of Goss grain clusters and surrounding matrix grains in primarily recrystallized (**a**,**c**,**e**) and annealed (**b**,**d**,**f**) silicon steel sheets. The regions of Goss grain clusters in primarily recrystallized sheets (marked as A, B, and C) and the identical region in further annealed sheets are depicted with black dashed lines. G1 and G2 are two grains with Σ5 and Σ9 boundaries between Goss grains, respectively.

The growth behavior of clustered Goss grains in terms of local size and grain boundary environment was characterized by in situ EBSD in three representative regions, and the result is shown in Figure 3. Low-angle and high-angle grain boundaries were, respectively, formed between Goss grains within clusters, and between Goss grains and adjacent matrix grains.

In region A with fine Goss grains (Figure 3a), adjacent matrix grains grew rapidly by consuming the clustered Goss grains by means of size advantages (Figure 3b). In region B with Goss grains larger than region A (Figure 3c), matrix grains did not have a sufficient size advantage to consume Goss grains and Goss grains survived during grain growth (Figure 3d). Goss grains in region C were similar in size to region B. However, there were two adjacent matrix grains, G1 grain (*φ*_1_ = 44°, *Φ* = 1°, *φ*_2_ = 53°) and G2 grain (*φ*_1_ = 41°, *Φ* = 60°, *φ*_2_ = 41°), having special grain boundaries with Goss grains (Figure 3e). The misorientations of G1 and G2 with respect to Goss grains were 39.7°~44.1°<001> and 36.1°~46.9°<110>, which were close to Σ5 (36°<001>) and Σ9 (~38°<110>). Both Σ5 and Σ9 had lower boundary energy [26] and higher mobility compared with general high-angle grain boundary [27,28]. During grain growth, Σ5 and Σ9 contributed to the rapid growth of the G1 and G2 grains at the expense of Goss grains. The G1 and G2 grains with a significant size advantage can further consume surrounding matrix grains (Figure 3f). The results demonstrate that grain clusters can markedly alter grain growth behavior and texture-differentiated GSD.

### 3.2. Grain Size Distribution

**Figure 4:**
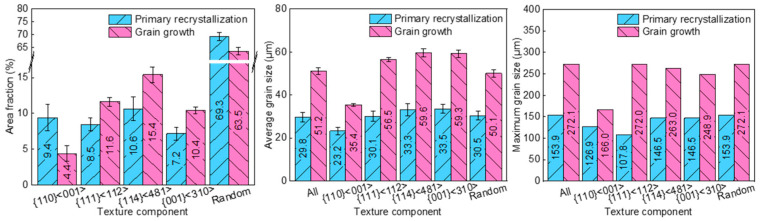
Area fraction, average grain size, and maximum grain size in primarily recrystallized and annealed silicon steel sheets.

Figure 4 shows the evolution of area fraction, average grain size, and maximum grain size. Goss grains had a size disadvantage at completion of primary recrystallization, and the average grain size (23.2 μm) as well as maximum grain size (126.9 μm) were over 20% smaller than other texture components. After grain growth, there was a notable reduction in the area fraction of Goss grains. Additionally, the ratio of average grain size of Goss grains to global average grain size was reduced from 0.78 to 0.69. The average grain size of primarily recrystallized {114}<481> and {001}<310> grains was, respectively, 33.3 μm and 33.5 μm, over 10% larger than the other grains. After grain growth, both area fraction and size advantage of {114}<481> and {001}<310> grains increased. The {111}<112> grains had a smaller maximum grain size (107.8 μm) before grain growth; the maximum grain size and area fraction of {111}<112> grains, however, exhibited a significant increase. Concurrently, the ratio of {111}<112> average grain size to global average grain size increased from 1.01 to 1.10, indicating a size advantage establishment during grain growth.

**Figure 5:**
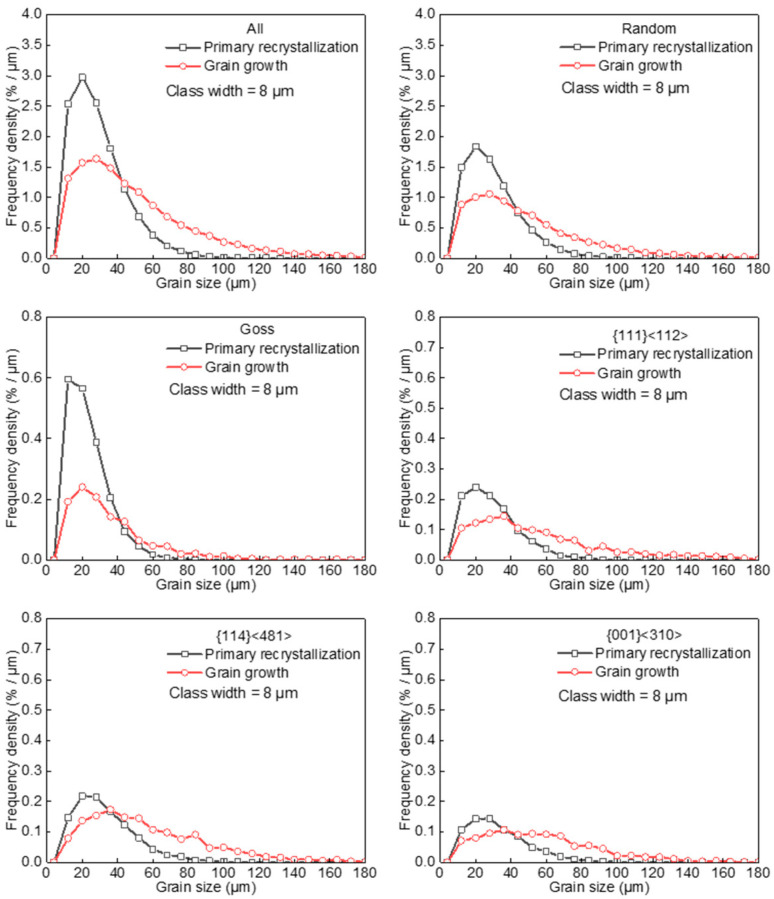
Grain size distribution in primarily recrystallized and annealed silicon steel sheets.

Figure 5 further illustrates GSD for major texture components. The Goss component exhibited a distinct GSD evolution of a slight shift toward large size side. This can be attributed to the significantly decreased frequency of small and medium Goss grains together with the slightly increased large Goss grains after grain growth. The {114}<481> and {001}<310> grains have an initial size advantage at completion of primary recrystallization, leading to an increase in area fraction and a significant shift in GSD toward large size side by grain growth. The {111}<112> grains had a similar initial average grain size and smaller GSD width compared with to randomly oriented grains. After grain growth, {111}<112> grains exhibited a significant frequency density increase in the GSD tail, consistent with the increase in average grain size, distribution width, and area fraction.

## 4. Local-Field Model for Grain Growth

The experimental results demonstrate that the heterogeneous spatial arrangement of grains leads to texture-differentiated GSD. In this study, we propose a local-field model to describe the growth behavior based on the local-field controlled grain growth kinetics.

### 4.1. Local-Field Model Framework

**Figure 6:**
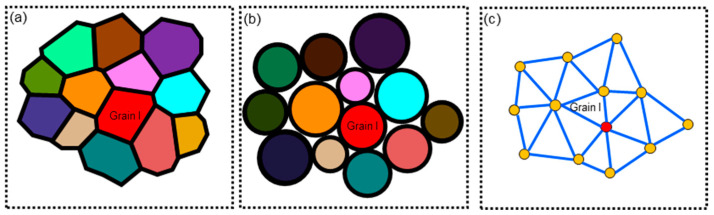
Representation of local-field model: (**a**) planar section of three-dimensional microstructure in polycrystalline materials, (**b**) planar section of three-dimensional microstructure with spherical grains, and (**c**) grain network in a planar section.

Each grain is embedded in a local environment, and the irregular shape of a grain is substituted by a sphere of equal volume, as shown in Figure 6a,b. During grain growth, a grain only exchanges matter with its adjacent grains, which allows the local-field model to capture the effect of spatial heterogeneity on grain growth. It is crucial to identify the adjacent grains of a grain at a given moment. For this purpose, the network analysis method, proposed by Dutra and Oliveira [29], was adopted to approximate the adjacency between adjacent grains. As shown in Figure 6c, the grains are abstracted as points, and the connection relationship is established by triangulating the points in space with each line connecting two adjacent grains. The position of a grain centroid is assumed not to shift during grain growth. At each time step, grains with nonpositive radii are removed and the network is reconstructed. Based on the above framework, adjacent grains of a grain can be traced.

### 4.2. Grain Growth Law Driven by Local-Field

Let us consider grain *I* adjacent to grain *J* (*J* = 1, 2, …) and they share the grain boundary *I*-*J*. The area of the grain boundary *I*-*J* can be expressed as [30]:

(1) $$A_{IJ}=\pi r_{IJ}^{2}$$

(2) $$r_{IJ}=\frac{R_{I}R_{J}(R_{I}+R_{J})}{R_{I}\sqrt{R_{I}(R_{I}+{2R}_{J})}+R_{J}\sqrt{R_{J}(R_{J}+{2R}_{I})}}$$

where${\;R}_{I}$ and $R_{J}$ are the radii of grain *I* and grain *J*, respectively. $\bar{\gamma^{I}}$ can be then expressed as:

(3) $$\bar{\gamma^{I}}=\frac{\sum_{J}A_{IJ}\gamma_{IJ}}{\sum_{J}A_{IJ}}$$

The motion velocity of grain boundary *I*-*J* ($v^{IJ}$) is [31]:

(4) $$v^{IJ}=m^{IJ}(\bar{\gamma^{I}}/R_{I}-\bar{\gamma^{J}}/R_{J})$$

where $m^{IJ}$ is the mobility of grain boundary *I*-*J*, $\bar{\gamma^{I}}$ and $\bar{\gamma^{J}}$ are average grain boundary energy of grain *I* and grain *J*, respectively. The growth velocity of grain *I* ($dR_{I}/\mathrm{dt}$) can be expressed as:

(5) $$dR_{I}/\mathrm{dt}=\frac{\sum_{J}A_{IJ}v_{IJ}}{\sum_{J}A_{IJ}}$$

The boundary energy (*γ*(*θ*)) and boundary mobility (*m*(*θ*)) are functions of boundary misorientation (*θ*) [32,33,34]. It is generally accepted that grain boundaries such as Σ5 (36°<001>), Σ9(~38°<110>), and Σ19a (27°<110>) exhibit a higher mobility and lower boundary energy [35,36] in silicon steel. Considering the effect of boundary misorientation and special grain boundary, the boundary energy and mobility can be expressed as [32,33,34,36]:

(6) $$\gamma =\left\{\begin{array}{c} \gamma_{c}\;\;\;\;\;\;\;\;\;\;\;\;\;\;\;\;\;\;\;\;\;\;\;\;\;\;\;\;\;\;\;\;\;\;\;\;\;\;\;\;\;\;\;\;\;\;\;\;\;\;\;\;\;\;\;\;\;\;\;\;\;\;\;\;\;\;\;\;\;\;\;\theta >\theta_{c}\mathrm{and}\omega k>\omega_{c}\\ \gamma_{c}\frac{\theta }{\theta_{c}}\left(1-\ln\frac{\theta }{\theta_{c}}\right)\;\;\;\;\;\;\;\;\;\;\;\;\;\;\;\;\;\;\;\;\;\;\;\;\;\;\;\;\;\;\;\;\;\;\;\;\;\;\;\;\;\;\;\;\;\;\;\theta \leq \theta_{c}\mathrm{and}\omega k>\omega_{c}\\ {\min[\gamma }_{c},\gamma_{c}\frac{\omega }{\omega_{c}}\left(1\;-\ln\frac{\omega }{\omega_{c}}\right)]\;\;\;\;\;\;\;\;\;\;\;\;\;\;\;\;\;\;\;\;\;\;\;\;\;\;\;\;\;\theta >\theta_{c}\mathrm{and}\omega k\leq \omega_{c} \end{array}\right.$$

(7) $$m=\left\{\begin{array}{c} m_{c}\;\;\;\;\;\;\;\;\;\;\;\;\;\;\;\;\;\;\;\;\;\;\;\;\;\;\;\;\;\;\;\;\;\;\;\;\;\;\;\;\;\;\;\;\;\;\;\;\;\;\;\;\;\;\;\;\;\;\;\;\;\;\;\;\;\;\;\;\;\;\;\;\;\;\;\theta >\theta_{c}\mathrm{and}\omega k>\omega_{c}\\ m_{c}(1-\exp(-5{\left(\frac{\theta }{\theta_{c}}\right)}^{4}))\;\;\;\;\;\;\;\;\;\;\;\;\;\;\;\;\;\;\;\;\;\;\;\;\;\;\;\;\;\;\;\;\;\;\;\;\;\;\;\;\;\;\theta \leq \theta_{c}\mathrm{and}\omega k>\omega_{c}\\ \max\left[m_{c},\;m_{\mathrm{spe}}\left(\exp\left(-{\left(\frac{\omega k}{\omega_{c}}\right)}^{2}\right)\right)\right]\;\;\;\;\;\;\;\;\;\;\;\;\;\;\;\;\;\;\;\theta >\theta_{c}\mathrm{and}\omega k\leq \omega_{c} \end{array}\right.$$

where *γ_c_* and $m_{c}$ are the grain boundary energy and mobility of high-angle grain boundaries, respectively. *θ_c_* is a cutoff angle set to 15° for high-angle grain boundary. *m_spe_* is the mobility of special grain boundaries, and ω is the deviation from a special boundary. *k* is a scaling parameter, and $\omega_{c}$ is the critical deviation angle. *k* = 1 and $\omega_{c}$ = 15° are normally adopted [36].

### 4.3. Calculation Process of Local-Field Model

First, the model reads a list of grains characterized by radius, orientation, and mass center coordinate, which can be provided by EBSD data or Voronoi tessellation. The grain growth occurs in three dimensions, even though their initial size and mass center coordinates are measured on two-dimensional sections; considering that the statistically measured sectional size can reliably approximate the three-dimensional size by adopting the same local grain size and grain boundary properties in three-dimension calculation as a two-dimensional measurement. Next, the time iteration loop is started. From time *t* to *t + dt*, the following sequence is executed:

(1) Determine the adjacent grains of each grain;
(2) Calculate the misorientation, and determine the grain boundary energy and mobility according to Equations (6) and (7);
(3) Determine the average grain boundary energy and mobility of each grain according to Equations (1)–(3);
(4) Calculate the radius increment for each grain using Equations (4) and (5), and update the grain size.

The present local-field model can be reduced to a mean-field model when it is assumed that a grain is randomly adjacent to all other grains. The grain boundary energy and mobility are generally defined as a function of misorientation, but the current model also allows grain boundary anisotropy to be assigned by specifying a list of grain boundary properties for flexibility.

## 5. Modeling and Analysis

In this section, GSDs were first calculated and compared with experiments to validate the model. Furthermore, texture-differentiated grain growth was modeled and analyzed by the constructed typical microstructural heterogeneity in textured materials.

### 5.1. Validation of Local-Field Model

Using EBSD data as input, the GSDs evolution with the mean-field model and the local-field model were calculated with $\gamma_{c}=1.11$ J/m^2^ [26] and $m_{c}\;$= 2.39 × 10^−13^ m^4^J^−1^s^−1^ [37] at 850 °C and $m_{spe}={10m}_{c}$ [36]. The time step (d*t*) needs to ensure that average radius increment is not greater than 1% for each step to control the error.

The calculation was terminated once the global average grain size agreed with the EBSD measurement, and the cumulative growth times were 581 s and 1047 s in the mean-field model and the local-field model, respectively. The cumulative growth time in the local-field model was much closer to the experiment (1200 s), suggesting that the present local-field model can capture grain growth kinetics more accurately.

**Figure 7:**
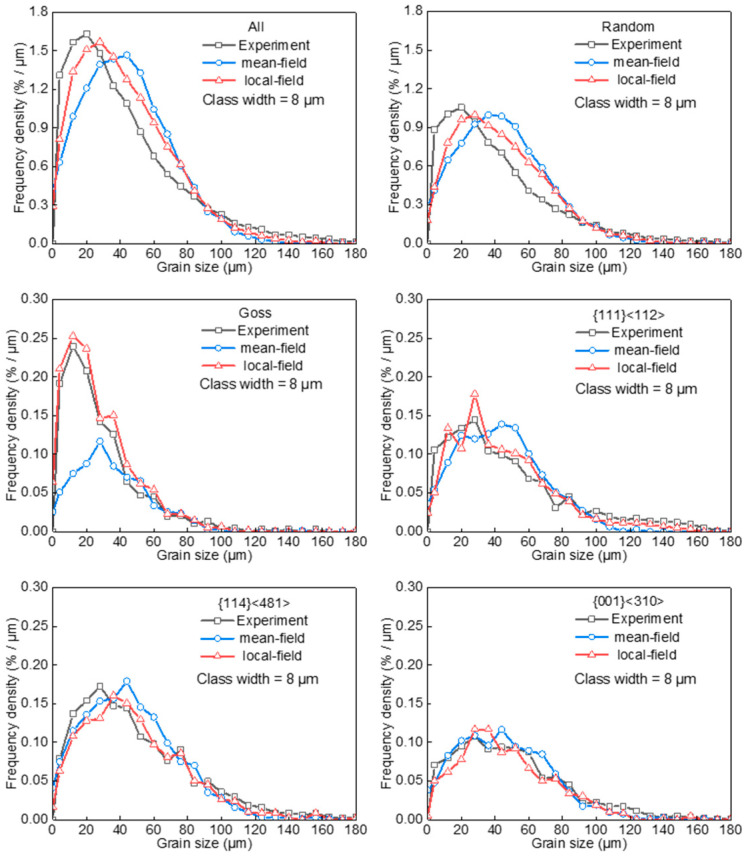
Measured and calculated grain size distributions for major texture components after grain growth annealing in silicon steel sheets.

Figure 7 shows the measured and calculated GSDs. Compared with measurements, the calculated frequency densities were lower in small and large size ranges and higher in medium size range. The GSDs calculated by the local-field model were evidently closer to the measured result, since the local-field model depicts the effect of heterogeneous spatial distribution on grain growth more accurately. With respect to Goss grains, which exhibit a higher degree of clustering and a size disadvantage, the mean-field model tends to underestimate the presence of low-angle grain boundaries surrounding the Goss grains and overestimates the number of large-sized grains adjacent to the Goss grains. The frequency density of Goss grains in the small and medium grain size ranges is thus significantly underestimated by the mean-field model, and the calculation deviation is narrowed efficiently by incorporating the effect of local environment. Therefore, the present model is more capable of capturing the evolution of texture-differentiated GSD than the conventional mean-field model.

### 5.2. Grain Growth within and out of Clusters

**Figure 8:**
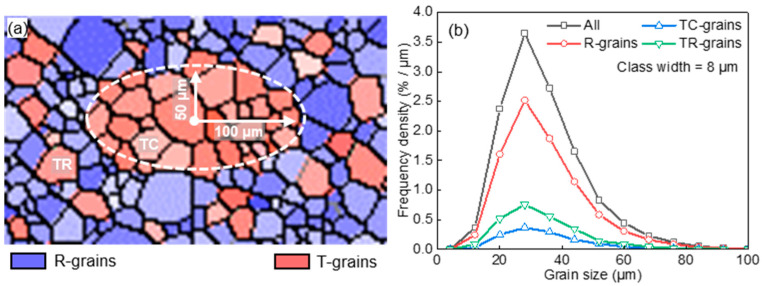
Schematic of initial microstructure (**a**) and grain size distribution (**b**) for the case of specially oriented grains clusteringly and randomly embedded in randomly oriented grains used in Section 4.2.

The heterogeneous spatial arrangement of grains with a specific orientation typically includes two types of morphology, i.e., clustered and randomly distributed microstructure. To model the distinct grain growth behavior within and out of a cluster, an initial microstructure (Figure 8a) was constructed by Voronoi tessellation, with specially oriented grains (T-grains) embedded in randomly oriented grains (R-grains). T-grains in a dense elliptical cluster and randomly embedded in R-grains are designated as TC-grains and TR-grains, respectively. Initial GSDs of TC-grains, TR-grains, and R-grains are shown in Figure 8b. Table 1 lists the microstructure information. The area fractions of TC-grains and TR-grains were 10% and 20%; T-T boundaries were low-angle grain boundaries with a misorientation angle of 5°; T-R and R-R boundaries were high-angle grain boundaries with a misorientation angle of 30°.

**Figure 9:**
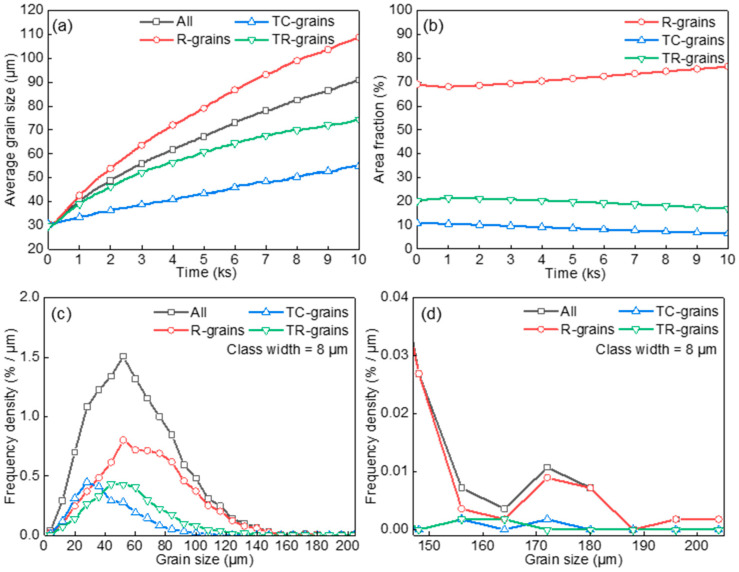
The evolution of average grain size (**a**), area fraction (**b**), and grain size distribution (**c**,**d**) for the case of grain clusters embedded in randomly oriented grains.

Figure 9a,b shows the evolution of average grain size and area fraction of TC-grains, TR-grains, and R-grains. Since T-T boundaries have much lower energy and mobility than R-T or R-R boundaries, the T-grains grow more slowly than R-grains, and also have strong resistance to aggression from R-grains in the early stages of grain growth. Moreover, due to the higher proportion of T-T grain boundaries, the growth rate of TC-grains is lower than TR-grains. As the grains continue to grow, T-grains were consumed by R-grains due to the size disadvantage. As presented in Figure 9c,d (*t* = 3000 s), the clustered spatial distribution enhances the effect of boundary energy and mobility anisotropy on grain growth. Although the peak position and distribution width were similar among TC-grains, TR-grains, and R-grains initially, their GSDs underwent a distinct change during further grain growth. The GSD of TC-grains exhibited a relatively smaller shift toward the large size side than TR-grains, while that of R-grains had a more significant shift toward the large size side. The contribution of TC-grains to the small-sized range (smaller than global average grain size) and the large-sized range (twice larger than the global average grain size) has been statistically analyzed. The proportion of TC-grains in the small-sized range increased more than 3 times (from 10% to 30.1%), while the proportion of TC-grains in the large-sized range decreased from 10% to 2.6%. This implies that a wide variety of texture-differentiated GSDs as well as global GSD can be modified by designing the spatial arrangement of various texture components in terms of the proportion of low-angle grain boundaries.

### 5.3. Grain Growth Affected by Special Boundary

**Figure 10:**
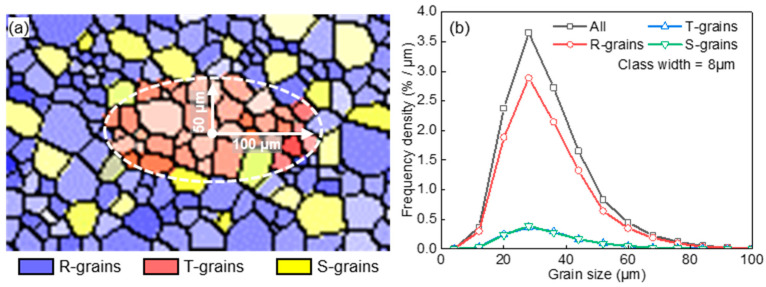
Schematic of initial microstructure (**a**) and grain size distribution (**b**) for the case of various spatial arrangements of grains and various grain boundary properties used in Section 4.3.

The growth behavior is also altered when special grain boundaries form between clustered grains and adjacent grains. An initial microstructure including Σ9 boundaries was constructed using Voronoi tessellation (Figure 10a), where clustered T-grains and randomly arranged S-grains were embedded in randomly oriented R-grains. Figure 10b shows the initial GSDs of different components. Table 2 gives the microstructure information. Initial area fractions of T- and S-grains were both 10%; T-S boundaries were Σ9(~38°<110>) boundaries with a deviation angle of 4°; T-T boundaries and S-S boundaries were low-angle grain boundaries with a misorientation angle of 5°; T-R, S-R, and R-R boundaries were high-angle grain boundaries with a misorientation angle of 30°.

**Figure 11:**
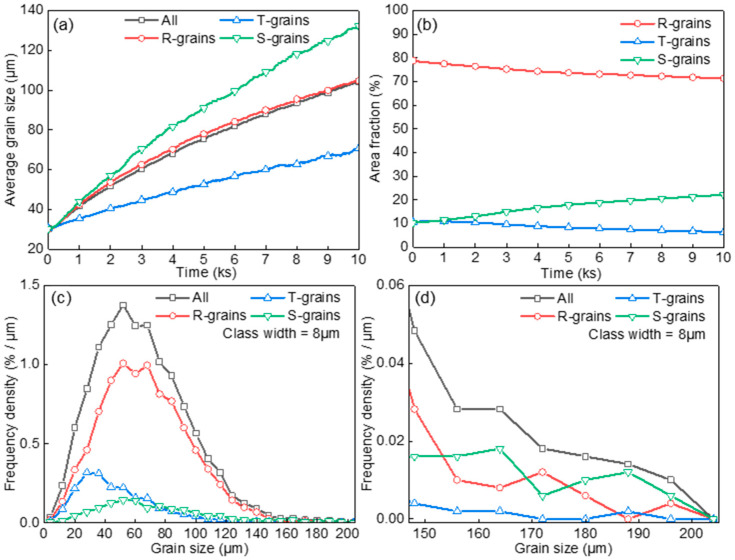
The evolution of average grain size (**a**), area fraction (**b**), and grain size distribution (**c**,**d**) in the case of various spatial arrangements of grains and various grain boundary properties.

Figure 11a,b shows the evolution of average grain size and area fraction of R-grains, T-grains, and S-grains. The S-grains had the highest growth rate and their area fraction increased by consuming R-grains and T-grains. This phenomenon can be attributed to the rapid growth of S-grains around T-grain clusters. As presented in Figure 11c,d (*t* = 3000 s), the GSD of S-grains exhibited a significant shift towards a larger grain side, in contrast to the slight shift towards a larger grain side for T-grains. It was found that although the total number of S-grains was less than R-grains, the number of large S-grains was more than R-grains. The proportion of S-grains in the small-sized range decreased slightly (from 10% to 9.6%), while the proportion of S-grains in the large-sized range increased more than 2.6 times (from 10% to 26.6%). The results demonstrate that T-grain clusters foster the growth of S-grains, leading to a high frequency density of GSD in the large size range.

The effect of T-grain clusters on S-grain growth depends not only on the boundary properties but also on the adjacency probability between T- and S-grains. As the adjacency probability increases, which may be caused by a change in spatial positions or an increase in volume fraction, clustered T-grains exert a more pronounced influence on the growth of S-grains. The GSD evolution of {111}<112> and {111}<481> grains in the experiment confirms the importance of spatial positions of various texture components. Both {111}<112> and {114}<481> grains form near Σ9(~38°<110>) grain boundaries with Goss grains, but Goss grain cluster only promotes the growth of {111}<112> grains. This is because of the neighboring position for recrystallization nucleation of {111}<112> and Goss grains, resulting in a higher probability of {111}<112> grains being adjacent to Goss grain clusters. Consequently, the spatial arrangement of various texture components is an important approach for regulating the GSD.

### 5.4. Texture-Differentiated Grain Growth

Grains with size advantage or low-energy grain boundaries tend to grow, and the growth rate is proportional to grain boundary mobility. Texture-differentiated grain growth can be qualitatively assessed by comparing the key factors including initial grain size, grain boundary energy, and grain boundary mobility among different texture components. Based on the above results, clustered grains with a sharp texture will exhibit a slower growth rate than those with a diffuse texture or randomly distributed grains due to the lower mobility of grain boundaries. Meanwhile, the grain growth out of a cluster is closely related to the grain size and texture in the cluster.

The quantitative analysis of texture-differentiated grain growth can be implemented by employing the present local-field model, which can precisely predict grain growth based on the initial heterogeneous microstructure and anisotropic grain boundary properties. In this work, a typical microstructural heterogeneity and grain clusters with specific orientations were considered. It is, however, worth mentioning that the effect of various spatial heterogeneities, such as grain size and grain boundary properties, on grain growth can also be effectively captured by the local-field model. Furthermore, grain boundary segregation and second-phase pinning can be incorporated in the model to study the effect of spatial heterogeneity from chemical composition or second-phase on grain growth.

## 6. Conclusions

1. Grain growth in Fe-3%Si steel exhibits an evident orientation dependence. The heterogeneous spatial distribution of Goss grains significantly alters the local grain size and grain boundary environment for grain growth, resulting in texture-differentiated grain growth and GSD evolution;
2. A local-field model is proposed by identifying the local grain size and grain boundary of each grain, which is capable of accurately describing texture-differentiated grain growth in the case of heterogeneous spatial distribution of various texture components;
3. The GSD evolution, after grain growth at 850 °C for 3000 s in microstructure with grain clusters embedded in randomly and specially oriented grains, can have an increased proportion of more than 3-times in the small-sized range due to survived clustered grains, and by more than 2.6-times in the large-sized range due to Σ9 grain boundaries between clustered grains and adjacent grains, respectively.

## Figures and Tables

**Table 1:** Grain orientation, spatial arrangement, and area fraction of various grains in the initial microstructure used in Section 4.2.

Grains	Grain Orientation	Spatial Arrangement	Area Fraction (%)
TC-grains	T-orientation	Cluster	10%
TR-grains	T-orientation	Random	20%
R-grains	Random orientation	Random	70%

**Table 2:** Grain orientation, spatial arrangement, and area fraction of various grains in the initial microstructure used in Section 4.3.

Grains	Grain Orientation	Spatial Arrangement	Area Fraction (%)
T-grains	T-orientation	Cluster	10%
S-grains	S-orientation	Random	10%
R-grains	Random orientation	Random	80%

## Data Availability

The datasets presented in this article are not readily available because the data are part of an ongoing study.
